# Pancreatic neuroendocrine tumors: the basics, the gray zone, and the target

**DOI:** 10.12688/f1000research.10188.1

**Published:** 2017-05-10

**Authors:** Dionysia Kelgiorgi, Christos Dervenis

**Affiliations:** 1 Department of Surgery, Euroclinic Athens, Athens, Greece; 2 Department of Surgical Oncology and HPB Surgery, Metropolitan Hospital, Athens, Greece

**Keywords:** panNETs, islet cell tumours, pancreatic neuroendocrine tumors

## Abstract

Pancreatic neuroendocrine tumors (PanNETs) manifest with a range of symptoms and pose a therapeutic challenge. A team approach, in which many specialists come together, is necessary in the quest for the best patient-tailored treatment. Disciplines such as oncology, surgery, basic science, endocrinology, radiology, and nuclear medicine need to work side by side, equally contributing to patient care and to advancing our better understanding of this fascinating disease.

## Introduction

Pancreatic neuroendocrine tumors (PanNETs) are rare neoplasms; however, they constantly intrigue the scientific community. For those who are familiar with PanNETs, the reason for this interest is their multi-faceted clinical presentation on one hand and their challenging treatment on the other. Even more so nowadays, clinicians need to become familiar with this pathologic entity, since there is an increase in both apparent incidence and survival, which in turn make these diverse neoplasms more prevalent ^1^. We will address two separate topics, which, in our opinion, are the most interesting ones in the overall management of these neoplasms. The first topic is the management of patients with small, asymptomatic lesions; the second one is the future of treatment for patients with more advanced disease.

## PanNETs: an overview

PanNETs were previously known as islet cell tumors, although it has been recently suggested that they may arise from pluripotent pancreatic cells of the ductal/acinar system ^2^. PanNETs comprise 1–2% of all pancreatic tumors and about 7–9% of all gastroenteropancreatic neuroendocrine tumors (NETs). PanNETs exhibit the most aggressive behavior among the latter ^3– 6^. Most PanNETs have a slow, indolent growth and are asymptomatic. As a result, the majority of patients are diagnosed at an advanced stage ^7^. The incidence of this disease is reported to have increased over the years, with an annual incidence of 0.22 per 100,000 in the USA, while similar annual incidences have been reported in Europe and Asia as well. Furthermore, this increasing incidence concerns not only panNETs but also all GEP-NETs, with as much as a 2.5-fold rise over a 15-year period. Much of this increased incidence is suggested to be the result of increased detection of asymptomatic lesions owing to the increased use of abdominal imaging ^5^. There seems to be a peak of incidence between the sixth and seventh decade of life, with a slight male predominance ^3, 8^.

PanNETs can be sporadic or part of hereditary syndromes including von Hippel-Lindau, neurofibromatosis, and, the most frequent of all, multiple endocrine neoplasia type 1 (MEN-1), which is an autosomal dominant disorder caused by a mutation on chromosome 11q13 ^9^.

A primary classification is based on the presence, or not, of secreted hormones and neuropeptides from the neoplastic cells, with associated clinical symptoms, thus distinguishing PanNETs as functioning (F-PanNETs) or non-functioning (NF-PanNETs). Depending on the hormone produced, different clinical manifestations arise. On the contrary, NF-PanNETs don’t produce hormones, or, if they do, those hormones have no clinical implication. When NF-PanNETs are symptomatic, they produce either non-specific symptoms or mass-related symptoms, the latter depending on the location and size of the lesion ( Table 1) ^10^. NF-PanNETs represent 60–90% of all PanNETs, while, amongst the F-PanNETs, insulinoma is the most common, followed by gastrinoma ^4^.

**Table 1.** Pancreatic neuroendocrine tumors (PanNET): related hormones and clinical presentation.

Functional pancreatic neuroendocrine tumors		
TUMOR	HORMONE	SYMPTOMS/SIGNS
Insulinoma	Insulin	Hypoglycemia
Gastrinoma	Glucagon	Peptic ulcer, diarrhea, gastroesophageal reflux disease
Glucagonoma	Glucagon	Necrolytic migratory erythema, diabetes, depression
Somatostatinoma	Somatostatin	Diarrhea, diabetes, hypochlorhydria, cholelithiasis
VIPoma	Vasoactive intestinal peptide	Watery diarrhea, hypokalemia, achlorhydria
Non-functioning PanNET	Pancreatic polypeptide, neurotensin, ghrelin	Mass-related (jaundice, pancreatitis)

As it is with all malignant diseases, the categorization of patients, the risk stratification, the grading, and the staging as well as the recognition of important prognostic factors are imperative in order to choose the best therapeutic approach and follow-up plan for each patient. The currently used classification and staging systems are those proposed by the World Health Organization (WHO), the European NET Society (ENETS), and the American Joint Committee on Cancer (AJCC). Grading in the WHO system, which is also used by the ENETS, is based entirely on the proliferation rate of the tumor and categorizes PanNETs into grade 1 well-differentiated PanNETs (mitoses <2/10 HPF and Ki-67 index <3%), grade 2 well-differentiated PanNETs (mitoses 2/10–20/10 HPF and Ki-67 index 3–20%), and grade 3 neuroendocrine carcinoma (mitoses >20/10 HPF and Ki-67 index >20%). Staging in both the ENETS and the AJCC is based on the tumor–node–metastasis (TNM) classification ( Table 2). These systems have been tested and validated for their prognostic value, and their adoption is now essential in order to improve the management of patients ^11– 14^. As a matter of fact, WHO classification as poorly differentiated neuroendocrine carcinoma has been reported to be the strongest predictive variable (hazard ratio [HR] 9.9, *p*<0.001), followed by TNM stage IV (HR 5.9, *p*=0.020) ^15^. Additionally, among the well-differentiated NETs, the Ki-67 index, a neoplastic proliferation index (the percentage of cells that stain positive with the Ki-67 antibody) with cut-offs at 3% and 20%, is an essential index of tumor grading. Those with Ki-67 <3% do better than those with Ki-67 between 3 and 20%, highlighting the importance of accurate tumor grading ^7^. Apart from those abovementioned factors, others have also been found to have a predictive value, such as lymph node status. Patients with metastases to lymph nodes have been found to have worse prognosis than those without. Several studies have reported that these patients have a statistically significantly shorter estimated mean survival time of 19 ± 5 months compared to patients with no tumor-infiltrated lymph nodes and with an estimated mean survival time of 108 ± 9 months ( *p*<0.001) ^16^. These patients also have a decrease in disease-free survival (DFS) (log-rank <0.0001) ^17^ and a shorter time to development of metachronous liver metastases compared to those without metastases ( *p*<0.001) ^18^. In addition, the survival of patients with surgically resected p-NETs is reported to be statistically associated with tumor dimension (<2 cm versus >2 cm), functional status, radical resection (yes versus no) ( *p*<0.05) ^14^. The more prognostic information we have, the better we can approach and understand the disease and thus the better we can approach and manage the patient.

**Table 2.** Pancreatic neuroendocrine tumors: WHO classification and TNM staging.

WHO 2010/ENETS grading				
Grade		Differentiation	Ki-67 index (%)	Mitotic count/10 HPF
G1 (low)		Well	≤2	<2
G2 (intermediate)		Well	3–20	2–20
G3 (high)		Poorly	>20	>20
**TNM staging systems for pancreatic neuroendocrine neoplasms according to** **AJCC and ENETS**				
		**AJCC**		**ENETS**
**T1**		Tumors limited to the pancreas, <2 cm		Tumors limited to the pancreas, <2 cm
**T2**		Tumor limited to the pancreas, >2 cm		Tumor limited to the pancreas, 2–4 cm
**T3**		Tumor extended beyond the pancreas, but not involving celiac axis or artery		Tumor extended beyond the pancreas, or invading duodenum or common bile superior mesentery artery duct
**T4**		Tumor involving celiac axis or superior mesentery artery		Tumor invading adjacent structures
**N0**		No regional lymph node metastases		No regional lymph node metastases
**N1**		Presence of regional lymph node metastases		Presence of regional lymph node metastases
**M0**		No distant metastases		No distant metastases
**M1**		Presence of distant metastases		Presence of distant metastases
		**AJCC**		**ENETS**
**Stage**	**IA**	T1 N0 M0	**I**	T1 N0 M0
	**IB**	T2 N0 M0	**IIA**	T2 N0 M0
	**IIA**	T3 N0 M0	**IIB**	T3 N0 M0
	**IIB**	T1–3 N1 M0	**IIIA**	T4 N0 M0
	**III**	T4 N0–1 M0	**IIIB**	Any T, N1 M0
	**IV**	Any T, Any N, M1	**IV**	Any T, Any N, M1

AJCC, American Joint Committee on Cancer; ENETS, European Neuroendocrine Tumor Society; HPF, high power field; TNM, tumor–node–metastasis; WHO, World Health Organization

From the diagnostic point of view, when patients present with symptoms indicative of an F-PanNET ( Table 1), laboratory tests which confirm the oversecretion of the specific pancreatic hormone should be performed. The measurement of circulating markers, such as chromogranin A (CgA), pancreatic polypeptide (PP), and pancreastatin has both a diagnostic and prognostic role for both functioning and non-functioning tumors ^6^. Nevertheless, histology is the diagnostic determinant, particularly the identification of neuroendocrine cells expressing CgA and synaptophysin, the neoplastic morphology, and the Ki-67 index ^19^. Once the diagnosis has been made, the next crucial step is to localize the primary tumor and to determine the extent of the disease by assessing the nodal status and/or metastatic spread ^20^. This can be achieved by imaging studies, which can be either morphologic imaging modalities (CT, MRI, or endoscopic ultrasound [EUS]) or functional (somatostatin receptor scintigraphy [SRS] or PET). Morphologic imaging is most widely used for the initial evaluation and staging of disease, whereas functional imaging is useful for both detection and prognostic evaluation and can also change treatment planning ^21^. EUS has emerged as an important tool not only for diagnosis but also for staging and prognosis. EUS has higher sensitivity compared to both CT and MRI (96.7% versus 85.2% and 70.2%, respectively) ^22^, while being able to detect lesions, even in cases when the CT study was negative for a primary lesion in the pancreas. Furthermore, additional information can be obtained by implementation of fine needle aspiration (FNA). EUS plays an increasingly valuable role not only in diagnosis but also in disease prognosis, all of which justifies the inclusion of EUS in the diagnostic work-up in all patients with a suspected PanNET ^23– 25^. Functional studies, on the other hand, take advantage of the fact that most PanNETs overexpress somatostatin receptors with high affinity for the somatostatin analogues (SSAs) octreotide and lanreotide. By radiolabeling those analogues, SRS or octreoscan reveals lesions which express somatostatin receptors, with diagnostic and therapeutic applications. Other promising functional studies include 18F-fluorodeoxyglucose (18F-FDG) PET/CT, which measures the metabolic activity of cancerous tissues and is mainly used for poorly differentiated neuroendocrine carcinomas, and 68Ga-DOTATATE PET/CT, which allows molecular imaging of NETs. Molecular imaging is reported to have high diagnostic sensitivity and specificity and is useful not only because of its ability to detect occult tumor lesions but also because it can, subsequently, identify those patients who will benefit from SSA therapy ^26– 28^.

Surgery is the cornerstone of PanNET treatment, since complete surgical resection stands as the only potential cure. An overall aggressive approach is often recommended, since it has been associated with improved survival ^29, 30^. A study published in 2016 reports a median survival of patients with metastatic disease undergoing resection of the primary site to be 65 months versus 10 months for those without resection ( *p*<0.0001) ^31^. In another report, patients with metastasized PanNETs who underwent palliative primary-tumor resection showed a significant benefit in both overall survival (OS) (HR of death=0.41, *p*<0.001) and cancer-specific survival (HR of death=0.41, *p*<0.001) ^32^. Less-invasive (laparoscopic resection) and parenchyma-sparing (enucleation and middle pancreatectomy) procedures have also been validated to be a safe choice for the surgical treatment of PanNETs ^33– 35^. On the other hand, in 2015, Lesurtel *et al*. conducted a systematic review of the literature and tried to issue practice recommendations for hepatic resection versus non-resectional liver-directed treatments in patients with potentially resectable neuroendocrine liver metastases. They didn’t identify any robust evidence that a liver resection was superior to any other liver-directed therapy in improving OS or progression-free survival (PFS). In cases where liver metastases are present but surgery is not practical, other liver-directed therapies can be used, like TACE (transarterial chemoembolization), TAE (transarterial embolization), and RFA (radiofrequency ablation), but there are insufficient data to recommended one over the other ^36^.

Systemic therapy can also be effective in the treatment of patients with advanced disease and consists of the following broad modalities: SSAs, chemotherapy, targeted therapy, and peptide receptor radionuclide therapy (PRRT). Systemic therapy is applied in case of metastatic and/or recurrent disease and it is aimed at prolonging survival, alleviating symptoms, and improving the quality of life. SSAs (octreotide long-acting release [LAR] and lanreotide) are often the first-line treatment for the symptomatic control of F-PanNETs with well-documented efficacy ^37^. In addition to their effectiveness in alleviating symptoms, SSAs can also have an anti-proliferative effect on both F- and NF-NETs, as it has been demonstrated by two randomized controlled studies (RCTs), the CLARINET and PROMID trials, which have yielded evidence of improved PFS. The PROMID trial, which tested octreotide LAR on mid-gut NETs, demonstrated a median time to tumor progression of 14.3 months in the treatment group versus 6 months in the placebo group ( *p*=0.000072). After receiving treatment for 6 months, 66.7% of patients in the octreotide LAR group and 37.2% of patients in the placebo group exhibited stable disease. Functionally active and inactive tumors responded in a similar way ^38^. Lanreotide, tested in the CLARINET trial, produced a 32.8-month estimate for median PFS in patients randomized to lanreotide versus an 18.0-month median PFS for placebo. In addition, for the subgroup of patients with PanNETs, the median PFS was 29.7 months (lanreotide group) versus 12.1 months (placebo). Furthermore, the long-term safety and tolerability profile of lanreotide was favorable during a median treatment duration of 40 months ^39^. Cytotoxic chemotherapy is a treatment option for those cases where SSAs fail or for high-grade (grade 3) tumors; however, chemotherapeutic toxicity limits their widespread use. Capecitabine, dacarbazine, 5-fluorouricil, streptozotocin, and temozolomide are mostly used as single-agent or combination therapies. The combination of streptozotocin/doxorubicin, streptozotocin/5-fluorouracil, and, lately, capecitabine/temozolomide has been considered to be effective in prolonging time to progression and median survival ^20^. PRRT targets the somatostatin receptors with radiolabeled SSAs, thus facilitating the application of localized cytotoxic radiation. ^90^Yttrium and ^177^Lutetium are the two radionuclides currently used. PRRT shows promise but has significant toxicity, mainly renal, and it is reserved for patients who are resistant to other treatments ^40– 42^. Targeted therapy will be discussed later on.

Several societies have issued guidelines for the management of PanNETs, accompanied by comprehensive therapeutic algorithms, which can aid clinicians in their day-to-day practice ^20, 37, 43, 44^.

## Small, asymptomatic, incidental

In the past few years, there has been a significant increase in the incidence of small and asymptomatic NF-PanNETs, which is presumed to be the result of the widespread use of high-resolution imaging techniques and endoscopic procedures in screening programs ^45^. However, there is no clear consensus on the treatment approach for small (≤2 cm) lesions, and the current treatment options are either surveillance or surgical resection ^43^. The literature reveals enough studies which have assessed the OS and DFS of patients with small PanNETs that were either observed or operated upon, but those studies are mostly small and retrospective series.

Several studies have supported the observation of small PanNETs. A matched case-control study was conducted in 2015 in which 104 patients were observed and matched to 77 patients who underwent surgery based on tumor size at initial imaging. In both groups, the lesion was <3 cm at initial imaging. The patients who were observed were followed for a median of 44 months. During the observation period, there was no change in the median tumor size (1.2 cm, *p*=0.7) and no development of locoregional or distant metastases. The patients who initially had surgery were followed for a median of 57 months; 6% of these patients had recurrence after a median of 5 years, and 25% of the patients who were observed had surgery in the end, mainly because of tumor growth, physician’s preference, and patient’s choice. The reported rate of complications for the resection group was 32%, with 16 patients presenting grade 3 complications. The authors suggest that long-term outcome may not be compromised by a delayed surgical resection. Additionally, they suggest that for selected patients with incidentally discovered, small, asymptomatic, and stable lesions, an initial observation approach is both reasonable and safe ^46^. Another, also retrospective study of 35 patients was published in 2016. Of those, 20 underwent operative resection (OG) and 15 were managed non-operatively (NOG), while the median duration of follow-up was 30 months. The median tumor size was 2.3 cm and 1.4 cm, respectively. In this study, there was no difference in metastases between the OG and NOG ( *p*=0.3891) and no difference in OS rates (100% versus 90%) ( *p*=0.4292) or in DFS rates (80% versus 85%) ( *p*=0.5337). In addition, no difference in tumor growth/recurrence was observed ( *p*=0.4497). Although the small sample size of this study is a limitation, the authors concluded that patients with small NF-PanNETs can be managed NOG and have similar outcomes of those treated OG by resection. Small tumors (<2 cm) in both the NOG and the OG subgroups did not develop metastases, did not grow, and did not recur, and there were no deaths over the 28 months of follow-up. The authors reported that the only adverse outcome that these patients experienced in their management was operation-related morbidity, which was 35% ^47^. Massironi *et al*. published the results of a prospective study, which, although it is a small series, is a valuable addition to the literature because of its prospective nature. A total of 51 newly diagnosed patients with PanNET were enrolled; every 3 months for the first year and then every 6 months, 15 patients with PanNETs ≤2 cm were intensively followed up (FU group). All patients were classed as TNM stage I, except for one (stage IIA). A total of 21 patients underwent surgical resection (SR group): two were at TNM stage I, nine were at stage IIA, one was at stage IIIB, and nine were at stage IV. A total of 15 patients received systemic therapy (ST group) because of advanced disease or contraindications to surgery: five were at stage IIA, two were at stage IIB, and eight were at stage IV. The patients were followed-up for a median 50 months. The 4-year survival rate in the FU, SR, and ST group was 100%, 90.5%, and 61%, respectively ( *p*<0.0001). The disease remained stable in all but one patient in the FU group, whereas six patients in the SR group and five in the ST group showed disease progression. In this study, nearly 50% of the SR group manifested post-surgery complications, of which five were mild (post-surgical infection), three were moderate (pancreatic fistula), and two were severe (severe hemorrhage) events, while six patients developed post-surgical endocrine insufficiency (i.e. diabetes). Data from this report suggest that a “wait-and-watch” approach to early stage small PanNETs may be a safe option for carefully selected small PanNETs ^48^. On the other hand, there are also reports that would support the routine resection of small, asymptomatic PanNETs. In a retrospective study, the characteristics, surgical outcomes, and survival of 139 patients who were surgically treated for incidentally discovered PanNETs were analyzed. Additionally, the results were compared with 30 patients who had resection for symptomatic NF-PanNETs. Three patients (7.7%) of a total of 39 with lesions ≤2 cm had recurrence or late metastases and died of their disease. The data from both groups were compared and were found to have no large difference regarding the patient’s age, tumor size, disease, or survival. This study suggests that even though small tumor size may be indicative of a more stable disease, it does not guarantee a good outcome. The 5-year and 10-year OS rates were 81.7% and 65.4%, respectively, in the symptomatic group compared with 82.8% and 65.1%, respectively, for patients with incidentally identified tumors ( *p*=0.27). According to the authors, incidentally detected, NF-PanNETs can display aggressive behavior and are potentially lethal lesions, even when small. The authors suggest that patients with incidentally discovered NF-PanNETs should undergo tumor resection and careful postoperative surveillance regardless of the tumor size, since there is no size cutoff beyond which malignant behavior can be safely excluded ^49^. Similarly, Birnbaum *et al*., after comparing a cohort of 108 patients, 65 (61%) of whom had incidentally diagnosed tumors, concluded that even though incidentally diagnosed NF-PanNETs are associated with less aggressive features compared with symptomatic lesions, benign behavior cannot always be guaranteed. Specifically, incidental tumors were more frequently <2 cm (65% versus 42%, *p*=0.019), stage T1 (62% versus 33%, *p*=0.0001), node negative (85% versus 60%, *p*=0.005), and grade 1 (66% versus 33%, *p*=0.0001). Pancreas-sparing pancreatectomies (enucleation/central pancreatectomy) were performed more frequently in incidental lesions (62% versus 30%, *p*=0.001). In this setting, surgical resection is recommended for most ^50^. Finally, data from the National Cancer Database were analyzed and produced evidence of an OS advantage for patients undergoing surgery for PanNETs ≤2 cm. In this report, 380 patients were studied; 81% had surgery and 19% were observed. The difference in 5-year OS between those groups was evaluated. The 5-year OS was 82.2% for patients who underwent surgery and 34.3% for those who were observed ( *p*<0.0001). Patients with localized PanNETs ≤2 cm had an overall advantage with resection compared to observation, a fact that was found to be independent of age, comorbidities, tumor grade, and treatment with non-surgical therapies ^51^.

Taken together, we can conclude that small, ≤ 2cm, NF tumors with a low Ki-67 index display less aggressive behavior. However, there is not enough evidence to clearly support the one approach over the other. Only prospective randomized trials can produce more concrete evidence on which approach is superior over the other, but PanNETs are relatively rare and such a trial is difficult to conduct. We believe that the key point lies in the rigorous selection of the patients who will be observed, those with low-grade, small tumors. They must be closely monitored and offered the choice of surgery early upon the detection of disease progression or if the patient chooses that approach over observation.

## Targeted therapy

There has been substantial progress in our understanding of the molecular mechanisms driving PanNETs, which in turn led to a big breakthrough in systemic therapy ^52^. The mammalian target of rapamycin (mTOR) is a serine/threonine kinase with a regulating role in cell growth. It also has an important role in transducing signals which are mediated through a different pathway, that of phosphatidylinositol-3 kinase (PI3K)/protein kinase B (AKT). Furthermore, the effects of a number of growth factors such as insulin-like growth factor-1 (IGF1) and vascular endothelial growth factor (VEGF) are mediated through mTOR. Mutations in genes coding for the members of the mTOR pathway and downstream activation of the mTOR pathway have been described in PanNETs. Genes such as phosphatase and tensin homolog ( *PTEN*) and tuberous sclerosis 2 ( *TSC2*) have been found to have mutations in PanNETs. In addition, the VEGF pathway is also activated in PanNETs. Taking advantage of the significant role of these molecular pathways on cell growth and proliferation, various inhibitor agents emerged as promising treatment options ^53– 56^. The most used agents are everolimus (an oral mTOR inhibitor), sunitinib (an oral multi-targeted tyrosine kinase inhibitor that is active against VEGFRs, platelet-derived growth factor receptors [PDGFRs], stem-cell factor receptor, glial cell line-derived neurotrophic factor, and FMS-like tyrosine kinase 3), and bevacizumab (a humanized monoclonal antibody against VEGF), all of which have been thoroughly tested for their efficacy.

RADIANT-3, an international, multicenter, randomized, double-blinded, phase III trial, enrolled 410 patients who had advanced, low-grade, or intermediate-grade PanNETs to receive everolimus at a dose of 10 mg once daily (207 patients) or placebo (203 patients). The median follow-up period was 17 months. The median PFS was reported to be 11.0 months with everolimus versus 4.6 months with placebo ( *p*<0.001). Those patients who, owing to the advanced stage of their disease, had a poor prognosis had a 65% reduction in the relative risk of progression with everolimus therapy as compared with placebo ( *p*<0.001); 34% of the patients in the everolimus arm were alive and progression-free at 18 months as compared with 9% with placebo. Drug-related adverse effects were reported, mainly in the everolimus group, and were mostly grade 1 or 2 (stomatitis, rash, diarrhea, fatigue, and infections). A few years later, data on the final OS was published. In the RADIANT-3 study, everolimus was associated with a median OS of 44 months in patients with advanced progressive PanNETs, while the historically reported median survival for this group is around 27 months ^52, 57^.

Sunitinib was also tested in an international, randomized, double-blinded, placebo-controlled phase III trial in patients with advanced, well-differentiated PanNETs; 171 patients were enrolled and randomized in a supportive care group and a sunitinib group with a 1:1 ratio. The primary end point of the study was PFS, while objective response rate, OS, and safety of sunitinib were studied as secondary end points. An improvement in PFS with sunitinib was indeed observed: 11.4 months as compared with 5.5 months with placebo ( *p*<0.001). Concerning the secondary end points, the median OS could not be estimated for either study group, while eight patients who received sunitinib had a confirmed tumor response (two had complete responses and six had partial responses), with an objective response rate of 9.3%. Adverse reactions were also reported to be more frequent in the sunitinib group but were mainly grade 1 and 2, with the most common being diarrhea, nausea, asthenia, vomiting, and fatigue ^58^.

In September 2016, Roviello *et al*. published the results of their meta-analysis on the available clinical data related to the clinical efficacy of targeted therapies in the treatment of advanced PanNETs. The report included 1,908 cases. Among these, 1,012 were in the targeted treatment arm and 896 were in the control arm. The pooled analysis of the use of target agents in PanNETs revealed a significant increase in PFS compared to the control group ( *p*=0.003). Moreover, OS and response rate were improved ( *p*=0.03 and *p*<0.00001, respectively). These findings provide adequate support for the routine use of targeted agents for the treatment of PanNETs ^59^.

However, despite the fact that PanNETs initially respond to targeted therapy, they develop resistance to that treatment. In this case, combination therapy is advocated. It has been hypothesized that resistance to everolimus is based on a reactivation of mTOR through the IGF1–IGF1R pathway and AKT. Since both mechanisms have been reported to be inhibited by octreotide, the combination of everolimus with a SSA seems logical ^60, 61^. Bajetta *et al*. published a phase II multicenter study testing the everolimus–octreotide LAR combination, concluding that it was active and well tolerated in patients with advanced PanNETs ^62^.

Another approach is the dual inhibition of the mTOR and VEGF pathways. Their simultaneous or sequential blockade is an interesting approach in the effort to overcome the resistance. In a two-stage, single-arm, phase II study by Hobday *et al*., the mTOR inhibitor temsirolimus and the anti-VEGF-A monoclonal antibody bevacizumab were tested. Patients with well-differentiated or moderately differentiated PanNETs and progressive disease by RECIST were identified. They were administered temsirolimus 25 mg intravenously once a week and bevacizumab 10 mg/kg intravenously once every 2 weeks. A total of 58 patients were enrolled, of which 56 patients were assessed for response. The response rate was 41% (23 of 56 patients) and exceeded by far the goal of a relative risk of 20% set initially by the researchers. PFS at 6 months was 79% (44 of 56). Median PFS was 13.2 months. Median OS was 34 months. Hypertension (21%), fatigue (16%), lymphopenia (14%), and hyperglycemia (14%) were the most frequently observed grade 3 to 4 therapy-related adverse events. These results indicate that this biologic combination may become an important addition to systemic therapy for PanNETs ^63^.

Novel targeted agents have not only provided new treatment options for patients but also broadened our understanding of the biology of these tumors. As newer agents are being tested and other pathways are being understood, it is imperative to plan the optimal sequential strategy. The identification of biomarkers, which will help us predict the response to targeted therapy and the nature of the targeted therapy itself, holds the future, as it brings us closer to a personalized, patient-tailored treatment.

## Conclusion

The diversity of PanNETs is noteworthy, and their management requires teamwork. Each patient is unique, and the best treatment is the one that addresses the unique aspects of each patient. There is still much to learn and explore in this intriguing disease, but the future looks promising.
